# Co-operation between Polymerases and Nucleotide Synthetases in the RNA World

**DOI:** 10.1371/journal.pcbi.1005161

**Published:** 2016-11-07

**Authors:** Ye Eun Kim, Paul G. Higgs

**Affiliations:** Origins Institute and Department of Physics and Astronomy McMaster University, Hamilton, Ontario, Canada; University of Texas at Austin, UNITED STATES

## Abstract

It is believed that life passed through an RNA World stage in which replication was sustained by catalytic RNAs (ribozymes). The two most obvious types of ribozymes are a polymerase, which uses a neighbouring strand as a template to make a complementary sequence to the template, and a nucleotide synthetase, which synthesizes monomers for use by the polymerase. When a chemical source of monomers is available, the polymerase can survive on its own. When the chemical supply of monomers is too low, nucleotide production by the synthetase is essential and the two ribozymes can only survive when they are together. Here we consider a computational model to investigate conditions under which coexistence and cooperation of these two types of ribozymes is possible. The model considers six types of strands: the two functional sequences, the complementary strands to these sequences (which are required as templates), and non-functional mutants of the two sequences (which act as parasites). Strands are distributed on a two-dimensional lattice. Polymerases replicate strands on neighbouring sites and synthetases produce monomers that diffuse in the local neighbourhood. We show that coexistence of unlinked polymerases and synthetases is possible in this spatial model under conditions in which neither sequence could survive alone; hence, there is a selective force for increasing complexity. Coexistence is dependent on the relative lengths of the two functional strands, the strand diffusion rate, the monomer diffusion rate, and the rate of deleterious mutations. The sensitivity of this two-ribozyme system suggests that evolution of a system of many types of ribozymes would be difficult in a purely spatial model with unlinked genes. We therefore speculate that linkage of genes onto mini-chromosomes and encapsulation of strands in protocells would have been important fairly early in the history of life as a means of enabling more complex systems to evolve.

## Author Summary

*Trans*-acting polymerases are cooperative, because they copy neighbouring strands, and do not copy themselves directly. Inaccurate replication creates parasitic strands that act as templates but not ribozymes. It is known that in spatially distributed models with slow strand diffusion, clusters of cooperating polymerases arise that can survive in the presence of parasites provided that the error rate is less than a maximum limit (the error threshold). In the RNA World, we envisage multiple types of ribozymes working together. We would like to understand how a multi-ribozyme system could evolve from a system with a single type of polymerase ribozyme. As a first step in increasing complexity, we consider a two-ribozyme system in which there is one polymerase and one nucleotide synthetase that produces monomers for use by the polymerase. We are particularly interested to find conditions in which the chemical supply of monomers is too low for the polymerase to survive alone, but the additional monomers created by the synthetase allow the two-ribozyme system to survive where the single-ribozyme system could not. There is then a selective force for increasing the complexity of the system. Here we show that spatial clustering is sufficient to allow cooperation and survival of systems of unlinked ribozymes with different functions. Clusters form in which synthetases form fringes around the polymerases. Survival of the two-ribozyme system depends on several factors. The strand diffusion rate must be slow enough for cooperative clusters to emerge. The replication rate of the polymerase must be comparable to that of the synthetase. The diffusion rate of the monomers must be neither too slow nor too fast. The model considers the most difficult case for cooperation–unlinked genes with no compartments. The sensitivity of the two-ribozyme system that we study here suggests that evolution of a spatial system with multiple unlinked ribozymes would become increasingly more difficult as the number of components increased, and suggests that linkage and protocells would need to evolve relatively early in the history of life.

## Introduction

The RNA world hypothesis proposes that in the early stages of life on Earth, RNA sequences acted both as genes and as catalysts [1–3]. The key molecule in the RNA World would be an RNA polymerase ribozyme that used another RNA strand as a template and synthesized the complementary strand to the template. A polymerase that could rapidly and accurately replicate a template of its own length would be able to sustain life in the RNA World. As each polymerase copies a neighbouring strand rather than copying itself, sustained replication of this type requires cooperation between a group of polymerases that copy one another. The behaviour of cooperative polymerases is fairly well understood from theoretical models, as we will discuss below. The aim of this paper is to understand how a replicating system based on cooperating polymerases could evolve additional functions.

Any kind of additional ribozyme with a function that supports the polymerase could potentially add to the system with a single polymerase. The most obvious second type of function to consider is a nucleotide synthetase that catalyzes synthesis of monomers used by the polymerase. The polymerase copies the synthetase sequences as well as other polymerase sequences. A polymerase can only survive alone if the monomer concentration produced by abiotic chemistry is sufficiently high. If a synthetase is also present, the monomer concentration will be increased, and the two ribozymes can potentially survive together in conditions where neither of them could survive alone. Here, we investigate under what conditions these two ribozymes can survive by mutual cooperation.

Experimental work has demonstrated that specific ribozoymes in the laboratory have many of the features that would be required to support an RNA World. Polymerase ribozymes have been gradually developed in the lab by *in vitro* evolution [4–9] and the maximum lengths of the templates that can be replicated are now around 200 nucleotides, which is close to the length of the catalyst. Sustained autocatalytic replication of RNAs has been demonstrated in the lab with ligases [10] and recombinases [11]. However, these systems require continued input of relatively long strands as substrates, rather than the single nucleotide substrates required by the polymerases.

In order for ribozymes to have arisen, the environment of the early Earth must have supported the synthesis of RNA strands by abiotic chemistry. Experiments that show RNA polymerization have been carried out using clay catalysts [12–14] and using wetting and drying cycles in the presence of lipid bilayers or ammonium chloride salt [15–18]. Wet-dry cycling seems to promote polymerization because polymer bond formation becomes thermodynamically favourable in the dry phase but continued polymer growth is limited by restricted diffusion. The wet phase permits repositioning of molecules and allows further polymerization to occur [19]. Partially-ordered structures of nucleotides sandwiched between lipid lamellae have been observed by X-ray scattering [20,21], which suggests that the lipid bilayers help to organize the nucleotides in a configuration that is favourable for polymerization.

Another key step necessary before the RNA World would be sequence replication via non-enzymatic template directed synthesis. This has also been observed in the lab to some extent [22–24]. Taken together, the experimental studies of ribozymes and prebiotic chemistry make it seem plausible that an RNA world could have existed on the early Earth. Here we investigate some of the problems that would be faced by RNA replicators from the standpoint of evolutionary theory.

Computational models [25–32] have been used to study the way a replicating catalytic sequence could emerge from the mixture of random sequences that would be created by prebiotic chemistry. In two-dimensional spatial models [27,28], an RNA polymerase can arise by a rare event and spread deterministically across the surface, even in the presence of other non functional RNA strands that act as parasites. When diffusion of the RNA strands is slow, clusters of polymerases form that cooperate with one another [3], whereas parasites would destroy the system if it were well mixed. The fact that spatial clustering promotes the survival of cooperating replicators is well known from a number of different types of evolutionary models [33–44].

Cooperation between ribozymes of different functions, including polymerases, nucleotide synthetases, and lipid synthetases, has been investigated in a series of papers by Ma and co-workers [45–48]. These papers use a fairly realistic but complex model with a large number of different rate parameters, whereas here we want to keep the model with as few parameters as possible, in order to facilitate a theoretical understanding of the effects of changing parameters. These papers also assume that the reactions of the RNA strands occur within protocells. However, if a replicating system can be initiated without cells, this seems simpler to begin with. It is possible to envisage a complex metabolism for RNA-based life controlled by many different types of ribozymes with different functions, all of which are copied by the same RNA polymerase. We would like to know how complex a system could become in absence of cells. It has also been suggested that ribozymes were packaged in inorganic compartments [49] prior to the origin of cells. There would have been slow diffusion and spatial clustering in such a system in the same way as for a two-dimensional surface model.

Our previous work [28] has considered the origin and spread of a polymerase in a mixture of random sequences that act as parasitic templates. In the scenario that we study here, we presume that the polymerase system already exists and we ask whether a nucleotide synthetase can add to this system. The synthetase potentially benefits the polymerase by creating additional monomers and increasing the replication rate, but it also places the burden of its own replication onto the polymerase. Thus the synthetase has the potential to be both a parasite and a cooperator. It is fairly easy to show that cooperation of these two types of ribozyme is not possible in a well-mixed model with no spatial structure. Firstly, a polymerase system alone is overrun by parasitic templates in the well-mixed case. Secondly, even if there are no parasites, then two functional ribozymes can only co-exist in the well-mixed case if they have exactly the same replication rate, which will not be true in general. In this paper, we show that in a spatial model with slow strand diffusion, stable coexistence of the two ribozymes is possible, even though they have different lengths and different replication rates. The cooperating system is stable even when inaccurate replication creates non-functional mutants of both the polymerase and the synthetase, provided the error rate is less than a finite error threshold.

## Results

### Model description

The model operates on a two-dimensional square lattice. Each site may either be empty (state 0) or occupied by a single RNA strand (states 1–6), as summarized in Table 1. A polymerase (state 1) is able to use a neighbouring strand as a template to produce a strand complementary to the template. A nucleotide synthetase (state 4) is able to produce monomers that can be used by the polymerase. The complementary strands to these ribozymes (states 2 and 5) are not functional as catalysts, but they are required as templates to synthesize the ribozymes. In addition, states 3 and 6 represent mutant sequences of the polymerase and synthetase that are non-functional. The lengths of the two ribozymes are *L*_*pol*_ and *L*_*syn*_, and the lengths of the complementary strands and the mutants are the same as the corresponding ribozymes. All mutant strands of a given length are equivalent in this model; therefore, it is not necessary to distinguish between positive and negative strands in the mutants.

**Table 1:** Key to the model.

State	Strand type	Colour	Strand length	Replication rate	Mutation probability
0	Vacancy	White	-	-	-
1	Polymerase	Red	*L*_*pol*_	*k*_*pol*_	*M*_*pol*_
2	Complement to Polymerase	Orange	*L*_*pol*_	*k*_*pol*_	*M*_*pol*_
3	Mutant Polymerase	Black	*L*_*pol*_	*k*_*pol*_	*-*
4	Synthetase	Blue	*L*_*syn*_	*k*_*syn*_	*M*_*syn*_
5	Complement to Synthetase	Light Blue	*L*_*syn*_	*k*_*syn*_	*M*_*syn*_
6	Mutant Synthetase	Green	*L*_*syn*_	*k*_*syn*_	*-*

In each time step, of length *δt*, each lattice site is visited in a random order. If there is a strand on this site, it has an opportunity to be a template for replication. A template can only replicate if it has a neighbour that is a polymerase and another vacant neighbouring site into which the new strand is placed. The rate of replication is inversely proportional to the length of the template strand, as explained in the Methods section. Replication of the ribozymes is by alternate plus/minus strand copying—a ribozyme creates a complement and a complement creates a ribozyme. At each replication, there is a probability of a deleterious mutation that creates a non-functional mutant instead of the complementary sequence. The mutation probabilities *M*_*pol*_ and *M*_*syn*_ will in general be different for the two ribozymes because the strands can have different lengths, and the fraction of point mutations that are deleterious may depend on the structure and function of a ribozyme. Reverse mutation from a mutant sequence to a functional sequence is assumed to be negligible. Further details of the replication procedure are described in the Methods section.

Each site also has a monomer concentration. It is assumed that monomers are continually produced and destroyed by abiotic chemistry, so that an equilibrium concentration is reached. When synthetases are present, there is a local increase in the monomer concentration (see Methods). The replication rate is proportional to the monomer concentration on the template site. Monomers diffuse between lattice sites at a rate proportional to the diffusion parameter *D*. Strands can also diffuse by hopping into a neighbouring vacant site at rate *h*. Strands are treated individually and strand diffusion is a stochastic event for each strand, whereas monomer concentrations are continuous variables and monomer diffusion is treated deterministically (see Methods). Strands also break down to monomers at a rate *u*.

In order to understand the behavior of this model when all six types of strands are present, we will build up to this case from simpler situations. Firstly, we consider the polymerase with its complement and mutant sequences in absence of the synthetase. Secondly, we consider the two ribozymes and their complements in absence of mutation. Thirdly, we consider all six types of strand.

### Polymerases alone

**Fig 1:**
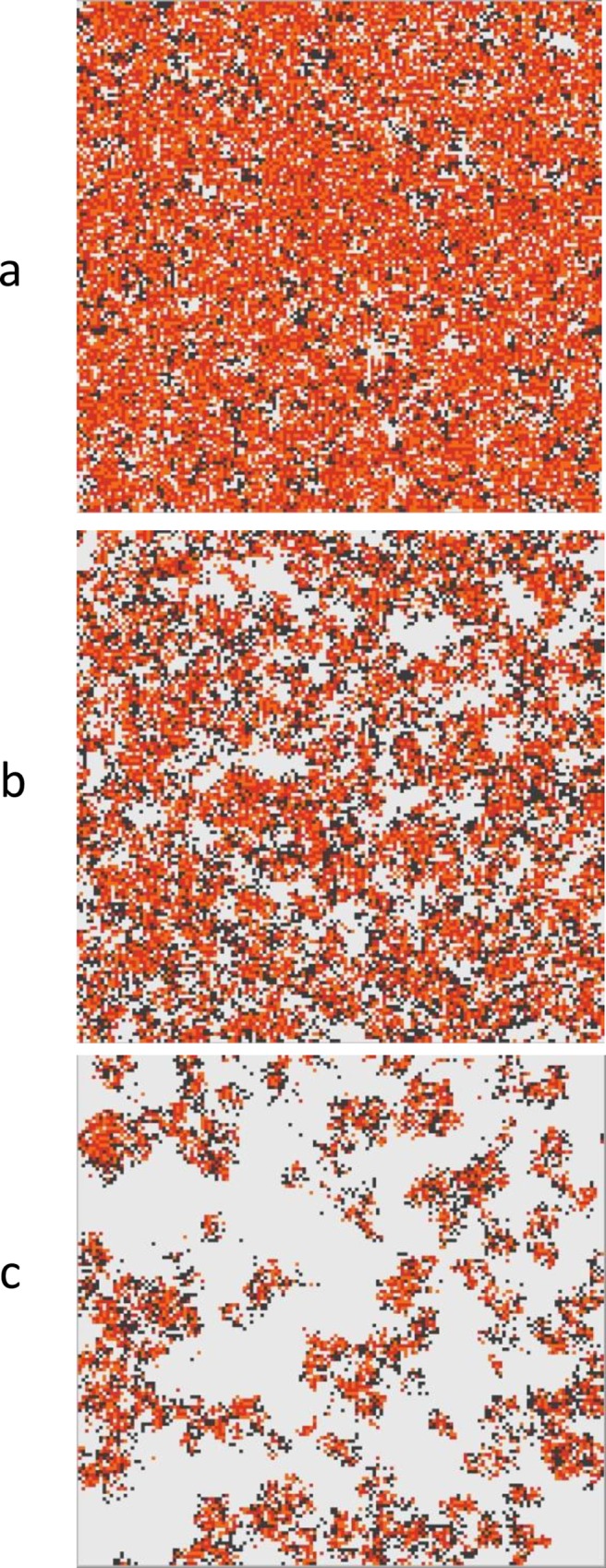
A system of polymerases, complements and mutants when *k* = 15, and *h* = 0, with three different values of the mutation probability (a) *M*_*pol*_ = 0.02; (b) *M*_*pol*_ = 0.08; (c) *M*_*pol*_ = 0.13. The colour scheme is explained in Table 1.

We first consider a situation with only polymerases and their complementary strands and mutants present (states 1–3). The only source of monomers is from abiotic chemistry, and we suppose that the monomer concentration *A* is fixed and constant everywhere in the lattice. Initially we set the hopping rate *h* to zero. This means that strands remain in the place where they are created. Clusters of strands nevertheless move across the lattice due to creation and destruction of strands. Typical equilibrium configurations are shown in Fig 1 for three different values of the mutation rate *M*_*pol*_. At low mutation rates, the polymerase and its complement occupy the surface fairly uniformly. As *M*_*pol*_ increases, the distribution becomes increasingly more patchy. The non-functional mutants are parasites of the polymerase. Spatial clustering of the polymerases and complementary strands allows them to survive even at relatively high mutation rates, whereas in a well-mixed case, where clustering cannot occur, the system is overrun by parasites for any non-zero mutation rate.

It should be noted that the polymerization rate constant, *k*_*pol*_, depends on the per-nucleotide polymerization rate constant *v*_*pol*_, the monomer concentration *A*, and the length of the strand *L*_*pol*_, as described in the Methods section. For the simulations in Fig 1, we fixed *L*_*pol*_ = 100 nucleotides, *A* = 500 nucleotides per site, and *v*_*pol*_ = 3, which results in *k*_*pol*_ = 15. However, since *A* is fixed and all the strands have the same length, it is only the combined value of *k*_*pol*_ that affects the simulation, rather than the three parameters separately.

**Fig 2:**
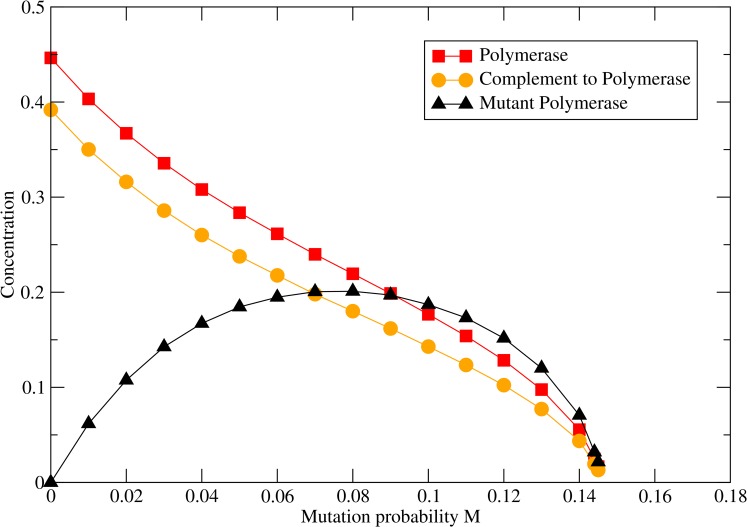
Concentration of strands as a function of mutation probability *M*_pol_ when *k*_*pol*_ = 15 and *h* = 0.

In Fig 2, time averaged strand concentrations are measured as a function of mutation rate with fixed polymerization rate. The concentrations of polymerase and complement decrease steadily with increasing *M*_*pol*_, while the mutant concentration passes through a maximum. At larger *M*_*pol*_ all three strand concentrations go to zero at the error threshold—the maximum mutation probability that is sustainable.

**Fig 3:**
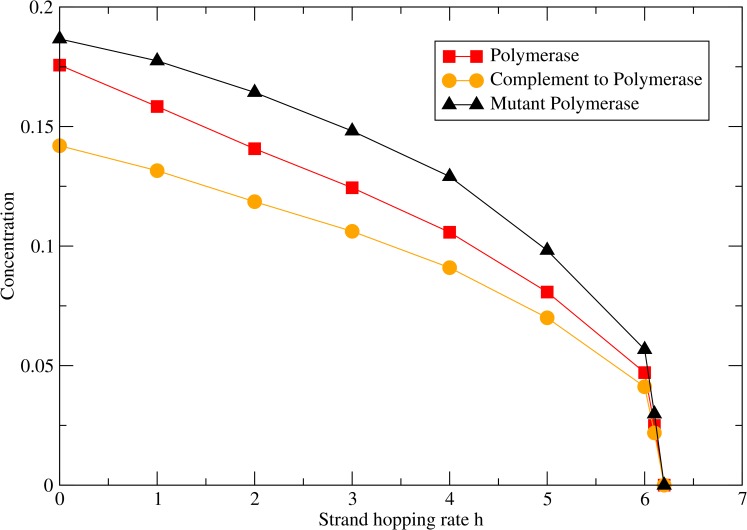
Concentration of strands as a function of strand hopping rate *h* when *k*_*pol*_ = 15 and *M*_*pol*_ = 0.1.

Another key parameter that affects the coexistence of the polymerase with the mutant strands is the strand hopping rate *h*. A small value of *h* allows the polymerases to form clusters where they replicate neighbouring strands. However as *h* increases, the clusters of polymerase start to disappear. There is increased mixing of polymerase and mutants, and mutants overrun the system until they eventually kill the entire population. This is shown in Fig 3.

The results in this section are very similar to those obtained in our previous paper [28] using a slightly different model. The previous paper used local rules that we called "Two's Company, Three's a Crowd". In that case, up to three strands were allowed on one lattice site. When a polymerase was on a site with one other strand, replication was possible, which produced a third strand on the same site. When there were three strands on a site, no further replication was permitted until one of the strands diffused away. In the model of the present paper, only one strand is permitted per site, and the template strand is on the neighbouring site to the polymerase, instead of on the same site. These two models are intended to represent the same situation. The well-mixed limits of these two models are the same (see the differential equations in the Methods section and in [28]). The results of the simulations of the spatial models are qualitatively similar: there is an error threshold at a finite value of *M*_*pol*_, and there is a maximum value of *h* above which mutants mix with polymerases and kill the system.

### Polymerases and synthetases with fixed monomer concentration and no mutation

Having understood the case with polymerases alone, we want to build towards the case of cooperation of polymerases and synthetases. As a next step, we will consider the case with polymerases and their complements (states 1 and 2), together with synthetases and their complements (states 4 and 5), but without mutant sequences, *i*.*e*. we will set both mutation probabilities *M*_*pol*_ and *M*_*syn*_ to zero.

When synthetases are present in the model it is necessary to specify the treatment of the monomers in more detail. We suppose that monomers are created chemically from precursors at a constant rate *a* per site and that they break down at a constant rate proportional to the current concentration on the site. Additionally each synthetase produces monomers at a rate *b*. If monomer diffusion is fast, the monomer concentration will be equal on all sites. The equilibrium monomer concentration is *A = a + bX*_*4*_, where *X*_*4*_ is the fraction of lattice sites that are synthetases (see Methods). The simplest case is where *b =* 0, and the synthetases are just non-functional parasites. We call these parasites 'independent' to distinguish them from parasites produced by mutation of the polymerase (state 3). If it can be shown that the polymerase can coexist with synthetases when they are just parasites (*b =* 0), then it seems likely that coexistence will also be possible when synthetases are functional (*b* > 0). Therefore we start with the *b =* 0 case.

**Fig 4:**
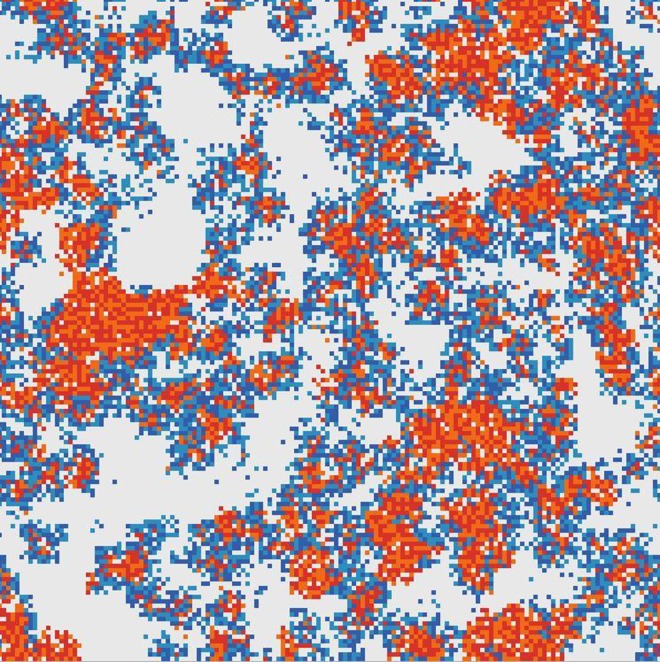
Polymerases with non-functional synthetases that act as independent parasites. *L*_*pol*_ = 100, *L*_*syn*_ = 50, *k*_*pol*_ = 25, *h* = 0, *M*_*pol*_ *=* 0 and *M*_*syn*_ = 0. The parasites coexist with the polymerases by forming bands around the fringes of the polymerase clusters. The colour scheme is explained in Table 1.

Fig 4 shows an example of coexistence of polymerases and independent parasites. We fix *A = a =* 500, *v*_*pol*_ = 5, and *L*_*pol*_ = 100, so that *k*_*pol*_ = 25, and consider varying lengths of *L*_*syn*_. The polymerization rate of a synthetase strand is *k*_*syn*_ *= v*_*pol*_*A/L*_*syn*_ *= k*_*pol*_*L*_*pol*_*/L*_*syn*_. Fig 4 shows a simulation with *L*_*syn*_ = 50, which means that *k*_*syn*_ = 2*k*_*pol*_. The parasites coexist with the polymerases despite the fact that they replicate twice as fast. The polymerases form clusters where strands are replicating their neighbours. The parasites can only survive on the fringes of these clusters as they need to be next to a polymerase in order to be replicated. The disadvantage to the parasites arising from spatial clustering counters the advantage they get from being shorter, faster replicators. A second possible outcome is that, if the parasite length is too long, the parasites multiply too slowly and they die out. The third possibility is where the parasites are too short. In this case, they multiply rapidly and kill the polymerases, after which they also die because they cannot replicate alone. There is thus a range of intermediate lengths of *L*_*syn*_ for which coexistence of the independent parasites with the polymerases is possible.

**Fig 5:**
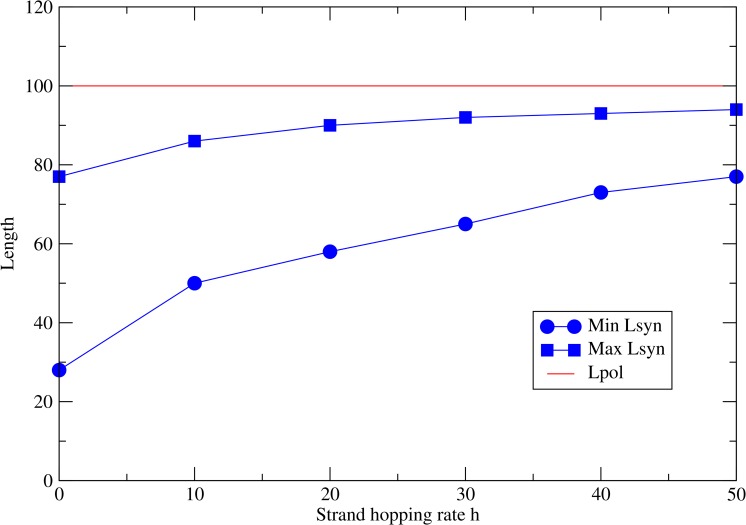
Coexistence of polymerases with independent parasites as a function of the strand hopping rate *h*. The red line shows the length of the polymerase *L*_*pol*_ = 100. The two blue curves show the upper and lower limits of *L*_*syn*_ for which coexistence is observed. The polymerization rate for the polymerase strands is fixed at *k*_*pol*_ *=* 25, and the rate for the parasites varies inversely with their length.

Fig 5 shows the boundaries of this region of coexistence. The width of the coexistence region is largest when the hopping rate is *h* = 0. As *h* increases, both the upper and lower length limits for coexistence increase and move towards the length of the polymerase, *L*_*pol*_ = 100. The limit of large *h* corresponds to the well-mixed case. Coexistence in the well mixed case is only possible if *L*_*syn*_ *= L*_*pol*_, as can be shown from the differential equations in the Methods section. As this will not usually be the case, we expect that coexistence of two different types of strands will not be possible in a well-mixed model. In the spatial model, however, there is a wide range of coexistence, especially when the hopping rate is small.

If we now consider the case of functional synthetases (*b* > 0), we see that it is essentially the same as the case with *b =* 0. If we determine the concentration of synthetases, *X*_*4*_, in a simulation with *b* = 0 and *a = a*_*0*_, then we can choose any other combination of *a* and *b* such that *a + bX*_*4*_ = *a*_*0*_, and the resulting equilibrium state should be the same. In particular, if we set *bX*_*4*_ *= a*_*0*_ and *a =* 0, this will also be the same as the original case where *a = a*_*0*_ and *b =* 0. This is significant because it means that the combination of the two ribozymes can survive together where neither can survive on its own. When *a* = 0, the only monomers are coming from the synthetase, so the polymerase cannot survive alone, and the synthetase cannot survive alone because it cannot replicate.

At this point we have shown that the spatial pattern that arises in Fig 4, where the synthetases surround the fringes of the polymerase clusters, allows both sequences to survive in parameter ranges where neither could survive on its own. We might be tempted to conclude that the problem of cooperation between ribozymes of different functions is solved. This conclusion would be premature, however, because we have only considered the case with zero mutation rate. It turns out that the model considered so far with fixed monomer concentration is not stable to mutations in the synthetase. For any non-zero rate of mutation in the synthetases, the functional synthetases are gradually replaced by mutant synthetases, so there is no further production of monomers by the synthetase. What happens then depends on the value of *a*. If *a* is large enough, the polymerases can survive by themselves, and the mutant synthetases are just independent parasites, as in the *b* = 0 case. If *a* is small, then the synthetases cannot survive alone, so the whole system dies out.

In this model, mutations in the synthetase behave in a different way to mutations in the polymerase. If *M*_*syn*_ = 0, then the cooperating system survives with non-zero *M*_*pol*_ up to a finite error rate. Spatial clustering prevents the invasion of polymerase mutants (as in Figs 1 and 2), but does not prevent the invasion of synthetase mutants. The problem is that if monomer concentration is equal everywhere, the mutant synthetases multiply just as fast as the functional ones, and deleterious mutations produce more and more mutants which eventually destroy the system. In order to get a stable coexistence between the polymerases and synthetases in the presence of non-zero mutation rates in both sequences, it is necessary to include spatial variation in the monomer concentration, as we do in the following section.

### Polymerases and synthetases (full model with monomer diffusion)

**Fig 6:**
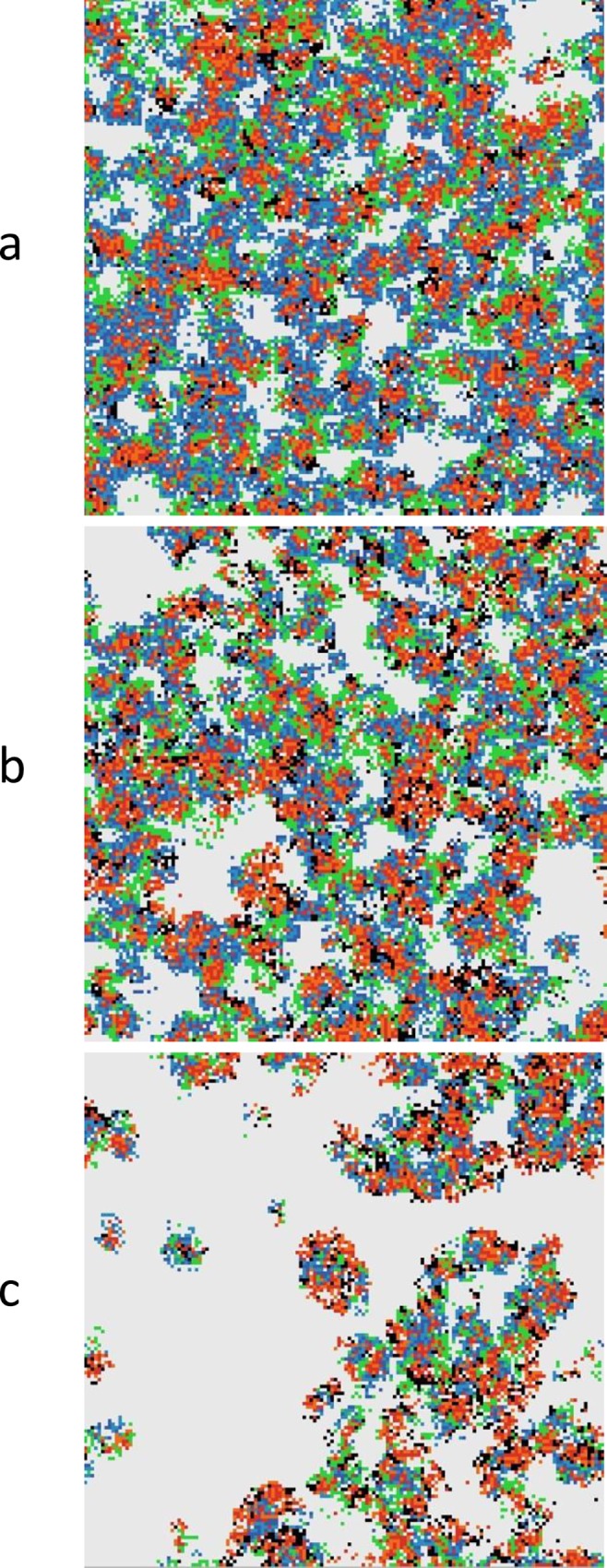
Coexistence of polymerases and synthetases in the model with full monomer diffusion. *L*_*pol*_ = 100, *L*_*syn*_ = 70, *a* = 0, *b* = 5000, *D* = 30, *v*_*pol*_ = 10. Three different values of mutation rate are shown (a) *M*_*pol*_ *= M*_*syn*_ = 0.02; (b) *M*_*pol*_ *= M*_*syn*_ = 0.04; (c) *M*_*pol*_ *= M*_*syn*_ = 0.065. The colour scheme is explained in Table 1.

**Fig 7:**
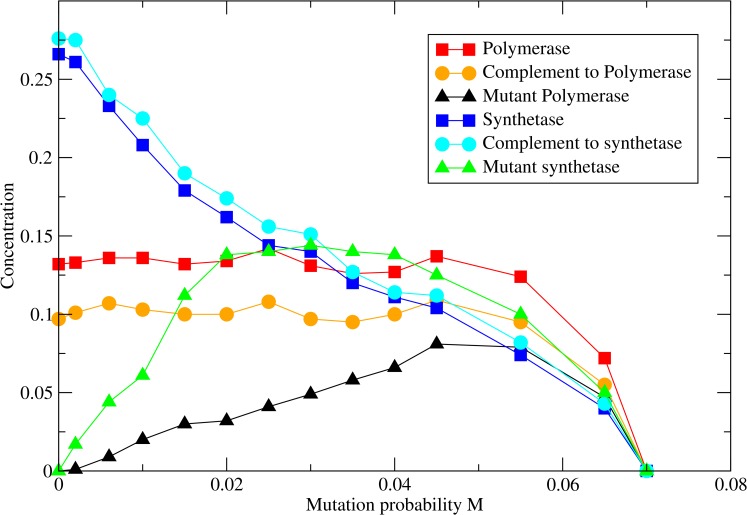
Concentrations of strand types in the full model with monomer diffusion as a function of mutation probability, with *M*_*pol*_ *= M*_*syn*_. Other parameters as in Fig 6: *L*_*pol*_ = 100, *L*_*syn*_ = 70, *a* = 0, *b* = 5000, *D* = 30, *v*_*pol*_ = 10.

We now consider the case where each synthetase produces monomers at rate *b* on its own site. There is a finite diffusion constant *D* for monomers, as described in Methods. In this case the system is stable to mutations in both polymerase and synthetase. In Fig 6 we show a case where *a* = 0 and monomers are only produced by the synthetase. Three different mutation rates are shown, with *M*_*pol*_ *= M*_*syn*_ in each case. Increasing mutation rate leads to increasing patchiness of the structure. Eventually an error threshold is reached. The concentrations of the strands are shown as a function of the mutation rate in Fig 7.

The monomer diffusion constant *D* is important in these simulations. If *D* is too small then the monomers accumulate on the sites of the synthetases and do not reach the sites occupied by the polymerases. This means that the replication rate of the polymerases becomes too low and the system dies out. If *D* is too large, monomers spread equally across the whole lattice, and this favours the multiplication of parasitic non-functional synthetases, as discussed in the previous section.

**Fig 8:**
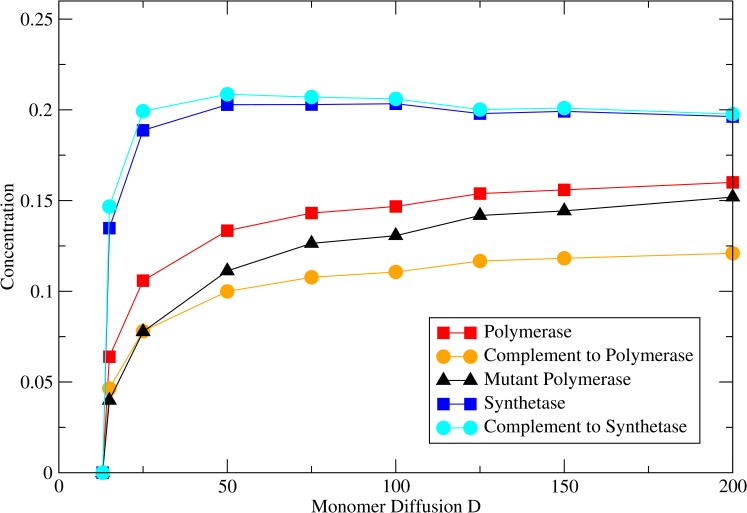
Strand concentrations as a function of monomer diffusion rate *D* when *M*_*pol*_ = 0.05 and *M*_*syn*_ = 0. Other parameters: *L*_*pol*_ = 100, *L*_*syn*_ = 70, *a* = 0, *b* = 5000, *v*_*pol*_ = 10.

**Fig 9:**
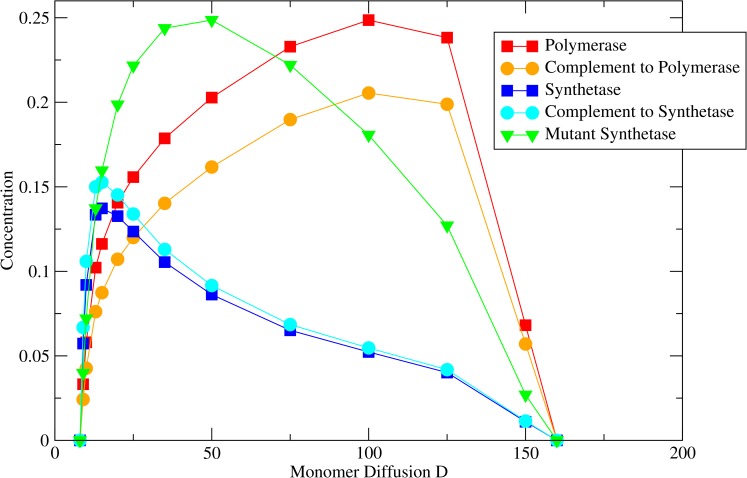
Strand concentrations as a function of monomer diffusion rate *D* when *M*_*pol*_ = 0.0 and *M*_*syn*_ = 0.05. Other parameters: *L*_*pol*_ = 100, *L*_*syn*_ = 70, *a* = 0, *b* = 5000, *v*_*pol*_ = 10.

To demonstrate that it is the mutant synthetases that kill the system at high *D* and not the mutant polymerases, we considered simulations where mutations occurred in either the polymerases or the synthetases but not both. In Fig 8, *M*_*pol*_ is fixed at 0.05, and *M*_*syn*_ = 0. If *D* is small, the system dies out because monomers do not reach the polymerases. At high *D* the system is stable and the concentrations tend to the values they have when the monomer concentration is equal everywhere. Fig 9 differs in that *M*_*syn*_ = 0.05 and *M*_*pol*_ = 0. In this case, the system dies at low *D* because monomers do not reach the polymerases, as before. However, at high *D* the system is again unstable because mutant synthetases out-compete the functional synthetases.

The rate of replication of any template strand is proportional to the monomer concentration on the site occupied by the strand. Monomers are synthesized on the sites occupied by the synthetases and diffuse outwards. The average concentration of monomers on synthetase sites is higher than on other types of sites. This gives functional synthetases an advantage over mutant synthetases, even though deleterious mutation is driving the increase of the mutant synthetases. This explains why the model with the full treatment of monomer diffusion is stable to mutations in the synthetase as long as *D* is not too large.

## Discussion

The idea of an error threshold, *i*.*e*. a maximum rate of deleterious mutations that can be sustained by a replicating system, is important in molecular evolution. The classical treatment of the error threshold problem [50] assumes that each strand has the ability to replicate via a 'one-molecule-makes-two' process. This is used as a model of virus replication [51,52]. It is assumed that the viral RNA is being replicated by a protein polymerase that is not itself evolving. The theory considers a 'master sequence' that replicates faster than the mutants. The concentration of the master sequence decreases as a function of the mutation rate and goes to zero at the error threshold—see, for example, Fig 4 of [50], which is similar to Figs 2 and 7 in this paper. The error thresholds in this paper occur for a different reason, however. Here, we are discussing a 'two-molecules-make-three' process, where the catalytic strand is part of the evolving system. The polymerase in our models is only partly analogous to the master sequence in the standard error threshold theory. Both theories assume that the functional sequence undergoes deleterious mutations to create non-functional sequences and that back-mutations from non-functional to functional sequences are negligible with respect to deleterious mutations. The neglect of the back mutations is reasonable unless the sequences are extremely short. It is this that leads to a sharp transition at the error threshold. However, the polymerase in our model differs from the master sequence in the classical theory in that it does not have an intrinsically faster replication rate. We have considered the case where the polymerase replicates all templates at the same per-nucleotide rate. For the polymerase to survive, it has to encounter other polymerases faster than it encounters mutant sequences. This is why spatial clustering is an essential part of the models discussed here but not a part of the classical error threshold theory.

Cooperative trans-acting polymerases have been studied in several kinds of models previously [27–28,34–35,42–44]. When there is a single kind of replicating molecule, is clear that spatial clustering is effective as a means of preventing the invasion of parasites. This paper extends the question to consider cooperation between independently replicating molecules with different functions. The conclusions of this paper are positive, in the sense that we have clearly shown that spatial clustering is also sufficient to allow coexistence and cooperation between two different ribozymes with complementary functions. However, we also think that these results are significant in highlighting some of the difficulties that will occur in evolving increased complexity of replicating systems of this kind. We would like to know how to get from a single-component polymerase system to an organism with hundreds of genes with different functions. We have treated a synthetase as a single ribozyme. However, synthesis of a relatively complicated monomer like a nucleotide presumably involves many steps, and four different nucleotides are required for RNA. It is possible to imagine a system involving multiple ribozymes with different functions that contribute to RNA synthesis. Only one of these sequences needs to be a polymerase, because all the other sequences can be replicated by the same polymerase. The results of the model studied here with just two functions lead us to a better appreciation of the difficulties associated with adding multiple functions. Spatial clustering is a simple mechanism that is sufficient to achieve a certain degree of cooperation; however, we doubt that the multiple-ribozyme system envisaged here could be achievable by this means alone. We have considered the most difficult case for cooperation—independent sequences with no compartmentalization. Organisms today go beyond this by linking genes on chromosomes and keeping their molecules together inside cells. The results in this paper suggest to us that it may have been necessary to evolve chromosomes and protocells rather early.

Firstly, in absence of compartments, spatial clustering arises only if we have limited strand diffusion. We have considered a 2d model with strands bound to a surface. Other systems with restricted diffusion might also work (*e*.*g*. small pores in rocks, or spaces between stacked lipid lamellae), but unrestricted diffusion in 3d will not. Given that clustering of polymerases is required for their own survival, it is necessary for other ribozymes with secondary functions to stay closely associated with the polymerase clusters. This gives rise to the interesting patterns of co-clusters seen in Fig 6. We have not yet attempted to develop a spatial model with more than two types of functional sequence, but we suspect that it would be difficult to achieve viable co-clustering patterns if there were more than a handful of essential types of genes that all need to be next to the polymerase in order to be replicated.

We focused on the case where *a* = 0 in Figs 6–9 because in that case it is clear that both polymerase and synthetase are essential and there is a mutually beneficial cooperation between the two. If *a* is sufficiently large, then the polymerase can survive by itself. A synthestase would then be an 'optional extra'. A new kind of functional gene must arise within a system that is already stable, *i*.*e*. a new gene must always be optional at the time and place where it first appears. If there were a multi-ribozyme system in which some of the genes were optional in this way, then it would not be necessary to have every type of gene present in every small neighbourhood. This would significantly reduce the constraints that would be necessary on the co-clustering patterns, and might make a diverse system with many gene types distributed across the surface easier to evolve. However, our experience with the current two-ribozyme model is that optional genes tend to do more harm than good. For example if *a* is sufficient to support the polymerase, and we add a functional synthetase (with *b* > 0), then the concentration of the polymerase tends to go down because of the extra load imposed by the synthetase. The polymerase would be better off on its own, and the synthetase is behaving like a parasite even though it has a beneficial function. Adding optional genes that are at least partially parasitic does not seem like a good general route towards evolving complex genetic systems.

Secondly, in absence of compartments, small molecule diffusion becomes an issue. This is illustrated in the present model by considering monomer diffusion explicitly. The model shows that the two-ribozyme system only survives if *D* is neither too large nor too small. In a multi-ribozyme system there will be many small molecules involved in the metabolism required for nucleotide synthesis. All of these will have to have appropriate diffusion rates. Compartments, such as lipid membranes in protocells, would solve the problem of keeping useful small molecules together close to the ribozymes that synthesized them. This would reduce the problem of invasion of mutant synthetases that we discussed above. Putting different kinds of sequences together in protocells also helps to favour cooperation, as is shown by stochastic protocell models [53–55]. A recent protocell model shows that large numbers of different replicator types can be maintained in protocells by the stochastic-corrector mechanism [56]. However, including cells also introduces complications associated with cell division, segregation of the genetic material between daughter cells, and transport of molecules across membranes. The origin of cells is a key early step in the evolution of life, but it is not yet clear just how early this step is. It is quite difficult to compare models of spatial clustering and protocells because they tend to be formulated in different ways, although see [43] for a good attempt at doing this.

It is possible that the first replicating sequences were already enclosed inside protocells almost by default. The recent experiments showing that RNA polymerization is facilitated by wetting and drying cycles in the presence of lipid bilayers [15–21] suggest that the first replicating polymers may have formed in the presence of lipids. This has led to the 'coupled phases' model for the origin of life [57] in which there is an alternation of replicating polymers from a dry phase between lipid lamellae to a wet phase encapsulated in protocells. If the lipids are there in large supply from the beginning, then the first replicators do not need to synthesize them, and if the membranes are continually breaking and reforming during the wetting and drying cycles, the replicators do not need to control the division of protocells or the transport of small molecules across the membranes. It would therefore be interesting to get a better understanding of whether a physical (non-living) process of encapsulation and dispersal can also create an environment in which cooperation of functional ribozymes can evolve.

Another feature of some surface-based models is that travelling waves of replicators and parasites can arise, such as the spiral waves seen in hypercycle models [33] and the more irregular travelling wave patterns seen in the replicase-parasite (RP) system described in section 5.3.1 of reference [44]. If short, rapidly multiplying parasites are added to our model with polymerases alone, we observe irregular travelling waves similar to the RP system [44]. A reviewer has suggested that cooperation of nucleotide synthetases and polymerases would be difficult in this model in the presence of short parasites because the synthetase may not be able to join the travelling waves of polymerases. This remains to be investigated in detail, but our preliminary results suggest that there are some cases where the joint system is stable in the presence of short parasites.

The evolution of linkage between genes of different functions is also a key step that we presume evolved rather early in life. If genes are linked, then the problem of different genes replicating at different rates (illustrated in Fig 5 in this paper) is solved, because they are forced to replicate together. Also, the problem of maintaining the spatial association of the two functions is solved because they are physically linked. The downside of linkage is that a longer strand with two genes would replicate more slowly than the shorter strands with a single gene. It was shown [58] that, in a protocell model, cells containing linked genes can out-compete cells containing separate genes because their progeny have a higher chance of inheriting a full gene complement. Selection on the cell can overcome selection for fast replication of individual genes. If there were just two ribozymes linked on a single RNA strand, it is possible that they would form two structural domains that individually fold to their functional structure at the same time, but this is difficult to envisage for more than a few domains. If there were many functional sequences linked on a longer chromosome, then it seems likely that some kind of separation of function between linked sequences (chromosomes) and individual sequences (catalysts) would have to arise. Thus the evolution of linkage would also create additional problems associated with control of transcription of individual genes and distinguishing transcription from chromosome replication.

In this paper we made the assumption that a replicating RNA system required an RNA polymerase ribozyme to catalyze sequence replication; hence we assumed that the first ribozyme was a polymerase and we considered the addition of secondary ribozymes to the polymerase system. This has been the traditional view of the RNA World community, and it has motivated the search for polymerase ribozymes by in vitro evolution. However, it is worth mentioning that sequence replication could in principle occur by non-enzymatic means [22–24], and if the rate of non-enzymatic replication were faster than the hydrolysis of the templates, a polymerase catalyst would not be necessary. This would open the way to considering alternative scenarios in which some other kind of ribozyme, like a nucleotide synthetase, came first [32,45,59].

A series of papers [38–41] using the metabolic replicator model (MRM) has studied the evolution of replicating ribozyme systems on mineral surfaces, and addresses questions that are very similar to those we have considered here. The MRM model is different from ours in important respects. In the MRM, there are several ribozymes of different types that produce small molecules that contribute to the replication process. The replication rate of a strand depends on the concentration of the different metabolic ribozymes within a local region called the metabolic neighbourhood. At least one ribozyme of each type must be present in the metabolic neighbourhood in order for strand replication to occur. The metabolic ribozymes in the MRM are similar to the synthetase in our models; however, we consider diffusion of monomers explicitly, whereas the MRM simply assumes that the small molecule products of the ribozymes extend uniformly to all sites within the metabolic neighbourhood. For the majority of the results in [38–41] it is assumed that replication is non-enzymatic (possibly because it is catalyzed by the mineral surface), and that there is no specific replicase ribozyme. A few results are given in [38] and [41] for a case where a replicase ribozyme (equivalent to the polymerase in our models) is included in addition to the metabolic ribozymes. They show that the replicase can promote replication beyond what is already given by non-enzymatic means; hence the replicase can be retained as a useful addition to the system. This scenario is different from the one we have studied, in which we assume that strands cannot replicate by non-enzymatic means. The polymerase is therefore essential in our model as a first ribozyme.

Studies with the MRM have shown that fairly large sets of metabolic replicators can be supported in surface-based replication systems [40–41] in which replication is non-enzymatic. As we have argued above, we suspect that building up larger sets of ribozymes will be more difficult in our model where the replication is controlled by the polymerase. Local contacts between neighbours are more crucial in our model than the MRM for several reasons. Nearest-neighbour contact is required between the polymerase and the strand being copied in our model. Nearest neighbour contacts are not required for the metabolic ribozymes in the MRM because their effect extends over the metabolic neighbourhood. We also assumed that a new strand can only be created if there is a vacancy on a neighbouring site to the template. The MRM model allows a new strand to be placed anywhere within a replication neighbourhood that can be broader than just nearest neighbour sites. Larger replication neighbourhoods allow coexistence of larger sets of replicators [40–41]. However, a large replication neighbourhood seems rather artificial. It would seem more reasonable to create the strand on the neighbouring site and then allow diffusion of the strand along the surface. Strand diffusion has different effects in the two types of models. In the MRM, strand diffusion is beneficial because it creates a more even mixture of ribozyme types, and this increases the rate of replication. Strand diffusion is detrimental in our model because it mixes the parasites with the polymerases and leads to loss of the polymerases (as in Fig 3 above). By incorporating the diffusion of monomers explicitly in our model we uncouple the motion of the monomers from the strand diffusion.

The above discussion emphasizes that determining the rate of non-enzymatic replication is a key issue for our understanding of the RNA world. Some degree of non-enzymatic replication is required to get the replication process started, because there must be at least one plus and one minus strand for a polymerase to begin with. The non-enzymatic rate need not be sufficient to sustain continued replication if the polymerase ribozyme is the first to emerge. The emergence of a polymerase from a random mixture is considered in our previous paper [28]. Once a polymerase system is established, catalytic replication is much faster than non-enzymatic replication, and the system survives even if the non-enzymatic rate is set to zero (as it is in the current paper). In the metabolic replicator scenario, in which metabolic replicators emerge before (or instead of) a polymerase, the rate of non-enzymatic replication must be sufficient to sustain replication at all times. A key issue is whether experimental conditions exist in which non-enzymatic replication is sufficiently fast and accurate to sustain replication of strands that are long enough to be metabolic ribozymes.

It is also worth noting that, although we have presented this paper in the context of the RNA world, the models are sufficiently general to apply to replication of any kind of nucleic acid analogue polymer system in which strands act as templates for synthesis of complementary strands.

In summary, there are several different ways in which co-operation at the molecular level is essential to the way the RNA world would have functioned [3]. In this paper we have focused on the issue of cooperation between unlinked ribozymes with different functions. The mechanism of spatial clustering, which arises when strand diffusion is very slow, such as when ribozymes are bound to a surface, is known to promote cooperation when there is a single type of polymerase ribozyme. Here we have shown that the same mechanism works to promote cooperation between a polymerase and a nucleotide synthetase. The model highlights several difficulties that must be overcome. The replication of the two kinds of sequences must occur at comparable rates. Co-clusters must arise in which the two types of sequences are closely positioned spatially. Diffusion of the monomers produced by the synthetase must be sufficiently fast to benefit the polymerases and sufficiently slow to prevent invasion of parasitic sequences. All these conditions can be satisfied within the two-ribozyme system that we studied. However, these difficulties are likely to be increasingly difficult to overcome as the number of independently replicating ribozyme components increases. Therefore we conclude that additional factors such as linked genes and protocells were probably necessary relatively early in the evolution of replicating systems.

## Methods

The model is simulated in discrete time steps of size *δt*. Strand replication, breakdown, and movement are treated as stochastic events that occur with a probability equal to the rate of that event multiplied by *δt*. For example, the time scale of the model is set relative to the strand breakdown rate *u* = 1; hence the probability of a strand breaking down to monomers is *uδt* per time step. A step size of *δt* = 0.002 was used in these simulations. In each time step, events occurred in the following order: replication and mutation; strand breakdown; monomer production and diffusion; strand hopping.

Replication and mutation—For each potential template strand, two *different* neighbouring sites are chosen randomly from the eight possible neighbours. Only if the first neighbour is a polymerase and the second neighbour is a vacancy, the template is replicated with a probability *kδt*, where *k* is the replication rate of the template strand, which is either *k*_*pol*_ or *k*_*syn*_ (see Table 1). If replication occurs, the new strand is placed in the vacant neighbouring site. The replication rates for the two types of sequences are

$$k_{pol}=v_{pol}A/L_{pol}\;,\;k_{syn}=v_{pol}A/L_{syn}\;,$$ (1)

where *A* is the monomer concentration, and *v*_*pol*_ is a constant derived below by considering a Michaelis-Menten reaction scheme. It is assumed that *v*_*pol*_ is a property of the polymerase and is the same for all templates. Under the approximations discussed below in the section on Michaelis-Menten kinetics, the replication rates are inversely proportional to the lengths of the templates.

In some of the simulations in this paper we assume that *A* is a constant that is equal on all lattice sites. The mean *A* is controlled by a balance of monomer production and breakdown back to precursors, as well as incorporation of monomers into strands and breakdown of strands back to monomers. In the fixed-*A* simulations, it is supposed that all these processes are in equilibrium and that diffusion of monomers is fast enough so that the same *A* concentration applies across the whole lattice.

In other simulations we treat *A* is a variable that is different on each lattice site. In the variable-*A* case, the replication rates (Eq 1) of each template depend on the *A* of the template lattice site. When a new sequence is created, the value of *A* on the template site is reduced by the number of monomers in the strand being produced (either *L*_*pol*_ or *L*_*syn*_).

When replication occurs, there is a mutation probability *M* that is either *M*_*pol*_ or *M*_*syn*_, depending on which strand is the template (Table 1). With a probability 1-*M* the new strand is the correct complementary sequence for the template. With a probability *M*, the new sequence is a mutant of the appropriate type. Reverse mutation from a mutant sequence to the corresponding functional sequence is assumed to be negligible. Secondary mutations in mutant sequences can be ignored because all mutant sequences are equivalent.

Strand breakdown—Once every strand has been given a chance to be a template, it has a chance to become degraded with probability *uδt*. This site then becomes a vacancy. The degradation rate *u* for all types of strands is assumed to be equal and is set to 1. In reality, degradation would involve multiple steps via fragments of shorter lengths. A longer strand would have more positions at which breakdown could start, but it would also take more steps to return to single monomers. The degradation rate should also be dependent on the structure of the strand. These are all complications that we have chosen to ignore in order to keep the model simple enough to be tractable. In the variable-*A* simulations, when a strand breaks down, a number of monomers equal to the length of the strand breaking down is added to the site previously occupied by the strand.

Monomer production, breakdown and diffusion—In the variable-*A* simulations, the concentration of monomers on each site is adjusted at each time step by an amount

δA=δt(a+bS−A), (2)

where *a* is the rate of production of monomers from precursors by abiotic chemistry, and *b* is the rate of production of monomers by synthetases. The variable *S* is 1 if there is a synthetase on the site, and 0 otherwise. The -*A* term is the rate of breakdown of monomers back to precursors. In the absence of replicating strands, the mean concentration is *A = a*. If the concentration of synthetases is *X*_*4*_ and the monomers are distributed uniformly across the lattice, the mean concentration is *A = a + bX*_*4*_.

When monomer diffusion occurs at a finite rate, *A* will vary between sites. Monomer diffusion occurs deterministically. At each time step a number of monomers *ADδt* leaves each lattice site and is distributed equally between the eight neighbouring sites. Each site also gains monomers from each of its eight neighbours. The net change in *A* on one site due to diffusion is

$$\delta A=D\delta t(-A+A_{neighbours}),$$ (3)

where *A*_*neighbours*_ is the mean concentration on the eight neighbours. In the fixed-*A* simulations, *A* is constant and the steps of monomer production, breakdown and diffusion are not necessary.

Strand diffusion—In each time step, each strand attempts to hop to one of its eight neighbouring sites with probability *hδt*. If the randomly chosen neighbour site is a vacancy, the strand moves to this site. It the neighbouring site is occupied, the strand stays where it is. We refer to strand diffusion as "hopping" in order to distinguish it from monomer diffusion.

Implementation—To get a visual understanding of the dynamical behaviour of the model, simulations were run using Netlogo [60]. The images shown in Figs 1, 4 and 6 use a lattice size of 150 × 150. The same model was also simulated in C in order to obtain numerical averages of quantities with a larger lattice size of 500 × 500. In both cases, periodic boundaries were used (*i*.*e*. the edges of the lattice are connected in a torus).

Well-mixed limit—If the hopping rate *h* is large, there is no correlation between the states of neighbouring lattice sites. This is the well-mixed limit in which we can write down deterministic differential equations for the fractions of lattice sites, *X*_*i*_, in each of the states *i* = 0–6 (Table 1). We will give these equations in order to make it clear how the spatial lattice model is related to the well-mixed case. It should be noted, however, that co-operation of the two types of ribozyme is not possible in the well-mixed case, for several reasons that we will explain below. Here we consider the fixed-*A* case only so that *k*_*pol*_ and *k*_*syn*_ are constants in the equations below.

$$\frac{dX_{1}}{dt}=k_{pol}X_{0}X_{1}X_{2}(1-M_{pol})-uX_{1}$$ (4)

$$\frac{dX_{2}}{dt}=k_{pol}X_{0}X_{1}^{2}(1-M_{pol})-uX_{2}$$ (5)

$$\frac{dX_{3}}{dt}=k_{pol}X_{0}X_{1}(X_{3}+M_{pol}X_{1}+M_{pol}X_{2})-uX_{3}$$ (6)

$$\frac{dX_{4}}{dt}=k_{syn}X_{0}X_{1}X_{5}(1-M_{syn})-uX_{4}$$ (7)

$$\frac{dX_{5}}{dt}=k_{syn}X_{0}X_{1}X_{4}(1-M_{syn})-uX_{5}$$ (8)

$$\frac{dX_{6}}{dt}=k_{syn}X_{0}X_{1}(X_{6}+M_{syn}X_{4}+M_{syn}X_{5})-uX_{6}$$ (9)

$$X_{0}=1-\sum_{i=1}^{6}X_{i}$$ (10)

It should be clear from this that the replication of type *i* depends on the product the template concentration *X*_*i*_, the polymerase concentration *X*_1_, and the vacancy concentration *X*_0_. In the lattice model, this corresponds to the assumption that the template must have a polymerase on a neighbouring site and a vacancy on a *different* neighbouring site. It should also be clear that mutations occurring on replication of type 1 and 2 strands, produce type 3 strands, while mutations in types 4 and 5, produce type 6 strands.

We will briefly summarize the key properties of these well-mixed equations. Firstly, if there is no synthetase present and polymerase replication is perfectly accurate (*M*_*pol*_ = 0), there is a stable solution for the polymerase with positive *X*_1_ and *X*_2_, provided the polymerization rate *k*_*pol*_ is sufficiently large. However, this state is unstable to mutation. For any non-zero value of *M*_*pol*_, the polymerase is overrun by its own mutations, and the system dies out. Secondly, if both ribozymes are present and replication is perfectly accurate (*M*_*pol*_ = 0 and *M*_*syn*_ = 0), then it is only possible for the two to coexist if *k*_*pol*_ *= k*_*syn*_. As this will never be exactly true for two ribozymes with different lengths and different structures, we conclude that it is not possible for two different types of ribozymes to cooperate in a well-mixed environment. In the lattice model with small *h*, spatial clustering arises that allows coexistence of the two ribozymes, as we show in the Results section.

Michaelis-Menten kinetics for replication—The synthesis of a complementary strand by a polymerase involves binding of the polymerase to the template and stepwise addition of monomers. For simplicity, we treat the synthesis of a new strand as a single step. Here, we calculate the effective rate of this single step by using the Michaelis-Menten enzyme kinetics model [61]. See also reference [42] for use of this scheme with replication dynamics. The reaction scheme is

$$T+X_{1}\begin{array}{c} \overset{k_{f}}{\to }\\ \underset{k_{r}}{\leftarrow } \end{array}C\overset{k_{cat}}{\to }T+T^{\prime }+X_{1},$$ (11)

where *X*_1_ is the polymerase, *T* is the template (any of the types of strand), *T’* is the complement to the template, and *C* is the complex of the polymerase and template. The rates *k*_*f*_ and *k*_*r*_ represent the binding and dissociation of the polymerase to the template, as in the standard Michaelis-Menten scheme. The rate of the catalytic step *k*_*cat*_ depends on the time taken for the individual monomer additions. If *v*_1_ is the rate constant for addition of one monomer, and the monomer concentration is *A*, then the mean time for a single addition is 1/*v*_1_*A*, and the mean time for synthesis of a strand of length *L* is *L/v*_1_*A*. We therefore approximate the catalytic step as a single step with rate *k*_*cat*_ *= v*_1_*A/L*.

Now, following the usual Michaelis-Menten method, we assume the complex is in equilibrium with the unbound polymerase. The total polymerase concentration is $X_{1}^{tot}=X_{1}+C$, and the complex concentration can be written as

$$C=\frac{k_{f}TX_{1}^{tot}}{k_{r}+k_{cat}+k_{f}T}.$$ (12)

The net rate formation of the complement *T’* is

$$k_{cat}C=\frac{k_{cat}k_{f}TX_{1}^{tot}}{k_{r}+k_{cat}+k_{f}T}\approx \frac{k_{cat}k_{f}}{k_{r}}TX_{1}=\frac{v_{1}k_{f}A}{k_{r}L}TX_{1}.$$ (13)

Above, we made the assumption that *k_r_* >> *k_cat_* + *k_f_T*, in which case $X_{1}\approx X_{1}^{tot}$. Under these assumptions, we see that the net rate of replication from a template of length *L* is *k*(*L*)*TX*_1_, where

$$k(L)=\frac{v_{pol}A}{L},$$ (14)

and *v_pol_* = *v*_1_*k_f_* / *k_r_*. In this paper, we considered strands of two lengths, *L*_*pol*_ and *L*_*syn*_, which gives the two replication rate constants in Eq (1) above.
